# Motor Protein Disruption Critically Alters Organelle Trafficking and Excitation–Contraction Coupling

**DOI:** 10.1523/ENEURO.0424-25.2026

**Published:** 2026-04-17

**Authors:** Hardik Bansal, Tadros A. Hana, Andrew H. Michael, Sevinch Kamaridinova, Jocelyn Bransford, Kiel G. Ormerod

**Affiliations:** ^1^Middle Tennessee State University, Murfreesboro, Tennessee 37132; ^2^Department of Neurosciences and Psychiatry, College of Medicine and Life Sciences, University of Toledo, Toledo, Ohio 43614

**Keywords:** *Drosophila*, motor protein, NMJ, trafficking

## Abstract

Trafficking of intracellular cargoes along the neuronal axonal microtubule tracks is a motor protein-dependent process. Here, we use a targeted genetic approach to knock down candidate kinesin genes involved in trafficking organelles in male and female *Drosophila melanogaster*. Live imaging experiments revealed intracellular trafficking changes, and kinesins 1 and 3 were identified as critical regulators. Disruptions in either gene product reduce rates of axonal trafficking in motor neurons (MNs) and lead to the formation of large intracellular aggregates. Kinesin disruptions led to significant changes in neuropeptide (NP) abundance at boutons and changes in synaptic morphology. Confocal imaging revealed fewer NPs trafficking through or getting captured by synapses in kinesin knockdown experiments and a dramatic reduction in NP release at MN terminals. A profound reduction in neuromuscular transduction and excitation–contraction coupling in kinesin 1 knockdowns, but not for kinesin 3, was observed. Collectively, the targeted genetic screen of kinesin proteins revealed disruptions in kinesin 1 and 3 greatly impact intracellular axonal trafficking. Taken together, several kinesins were identified which critically regulate organelle trafficking, and genetic disruptions in key kinesins also revealed critical disruptions in cellular morphology, function, physiology, and behavior.

## Significance Statement

Intracellular trafficking of cargo is a vital process critical to the survival of most cells. The functional cells within the nervous system, in neurons, are particularly dependent on trafficking given their profound energy consumption and need to transport materials over long distances, often exceeding 1 m. Here we conduct a targeted genetic screen to identify motor proteins involved in trafficking cargo in motor neurons. We reveal two genes previously identified in kinesin 1 and 3, critically involved in cargo transport. Our data show profound reductions in intracellular trafficking, and genetic disruptions lead to massive intracellular aggregations. Using cutting-edge approaches, our investigations uniquely show associated downstream impairments in neuromuscular junction structure, function, physiology, development, and behavior.

## Introduction

Motor proteins are critical for transporting intracellular cargoes over long distances using microtubule-assisted tracts along neuronal axons (Kurd and Saxton, 1996). Disruption in this transport system can impair neuronal development and function (Gunawardena and Goldstein, 2001; LaMonte et al., 2002). Neurons uniquely possess axons that can span long distances within the central and peripheral nervous system, which can exceed a meter in length in humans (Chew et al., 2012). While most cellular components are biosynthesized in the soma, it only comprises 1% of the cell volume, while the axon often comprises over 90% of the total cell volume (He and Jin, 2016). Furthermore, intracellular constituents transport bidirectionally, transporting material to synaptic terminals and subsequently back to the soma, doubling the demand on transport of some cargo (Weiss et al., 2012; Weiss and Littleton, 2016). Consequently, the role of trafficking is critical, accentuated by disruptions in axonal transport leading to debilitating diseases such as Alzheimer’s, Charcot–Marie–Tooth, and other neurodegenerative diseases (Hirokawa and Takemura, 2003; Hirokawa and Noda, 2008; Hares et al., 2017).

Motor proteins are key regulators of long-distance transport in axons (Hollenbeck and Saxton, 2005). They require microtubule tracts to move along the axons through an enzymatic process requiring hydrolyzation of ATP in motor-binding domains (Hollenbeck and Saxton, 2005). In axons, the plus end of microtubules is oriented toward the periphery of the neuron, near axon terminals. Kinesins travel toward the plus end of the microtubules allowing anterograde transport of cargoes. In contrast, dynein and dynactin mediate the minus-ended transport of cargoes, moving cargo toward the cell soma from axon terminals (Hirokawa and Takemura, 2005; Hollenbeck and Saxton, 2005). Considerable structural and functional variability exists within molecular motors like kinesin, unsurprisingly as >45 genes that encode for kinesin superfamily proteins (KIFs) are found in humans, classified into 15 kinesin families (Hirokawa, 1998; Miki et al., 2001; Lawrence et al., 2004; Hirokawa et al., 2009; Seal et al., 2023). Structurally, kinesins can be broken down into three main domains, a motor domain for microtubule binding via ATP hydrolysis, a dimerizing stalk domain for structural support, and a cargo-binding domain. Variability in the stalk and cargo-binding domains enable different kinesin family members to transport different cargo and interact with countless other proteins which can modify their transport speed, accessibility, and mechanochemical properties. Emerging evidence exists to demonstrate that recruitment of different subtypes of kinesins may be organelle or even cargo-specific (Yonekawa et al., 1998; Pilling et al., 2006; Barkus et al., 2008; Guardia et al., 2016; Lim et al., 2017; Hana et al., 2024).

Kinesin proteins are involved in numerous intracellular processes including microtubule dynamics, chromosome alignment, spindle formation, kinetochore assembly, endosome and organelle sorting, and microtubule-based organelle transport (Okada et al., 1995; Yonekawa et al., 1998; Pilling et al., 2006; Lipka et al., 2016; Lim et al., 2017). A subset of kinesins, e.g., kinesins 1, 2, and 3 (Kif1A, Kif1B), are known to transport a wide variety of cargo, but most research has focused on trafficking of synaptic vesicles (SVs), dense core vesicles (DCVs), and mitochondria (Pilling et al., 2006; Barkus et al., 2008; Kern et al., 2013; Guardia et al., 2016; Lim et al., 2017; Liao et al., 2018). Loss of kinesin 1 disrupts SV transport, causing vesicles to accumulate in the soma rather than reaching synaptic terminals; mutations in KIF1A, a kinesin 3 family member, result in defective anterograde transport and synaptic dysfunction (Okada et al., 1995; Yonekawa et al., 1998; Lipka et al., 2016). Similarly, DCV transport is impaired by KIF1A knockdown, leading to vesicle accumulation in proximal neuronal regions and reduced neuropeptide (NP) delivery to axon terminals (Barkus et al., 2008; Stucchi et al., 2018). In *Caenorhabditis elegans*, mutations in unc-104, a kinesin 3 family motor and *KIF1A* homolog, cause DCVs to stall in the soma rather than reach the synapse (Hall and Hedgecock, 1991; Zhang et al., 2017). Mitochondrial transport is also dependent on kinesin, as disruptions in kinesin 1 function lead to mitochondrial clustering in the soma and depletion from axons and dendrites, ultimately affecting energy distribution, redox regulation, calcium homeostasis, and other mitochondrial functions within neurons (Kurd and Saxton, 1996; Pilling et al., 2006). While these studies demonstrate clear defects in intracellular trafficking, the full molecular, cellular, and physiological consequences of these impairments remain incompletely understood.

Here we conducted a targeted genetic screen using RNAi to knockdown six families of kinesin proteins in *Drosophila* which aims to identify those involved in NP trafficking in motor axons. In this study, we also aim to examine the downstream molecular, cellular, and physiological impacts of disrupted motor protein trafficking. Disruptions in expression in two kinesin families, kinesin 1 and kinesin 3, caused a dramatic effect on NP transport. Live-axonal trafficking experiments reveal both kinesins 1 and 3 are involved in anterograde transport of NPs, while only kinesin 3 disruption impaired retrograde transport. Kinesin 1 knockdown caused numerous severe effects on neuromuscular junction (NMJ) development, synapse morphology, and intrasynaptic function/localization of NPs. Several physiological and behavioral parameters, including excitatory junctional potentials (EJPs), muscle force production, and larval crawling, were all critically reduced in kinesin 1 knockdown animals, while no significant phenotype was observed for kinesin 3. These findings highlight the important role of kinesin 1 in NP transport and NMJ development, whereas kinesin 3 primarily contributes to NP trafficking, particularly in retrograde transport, with minimal to no impact on synaptic development and motor behavior_._

## Materials and Methods

### Fly stocks and husbandry

*Drosophila melanogaster* flies were cultured on standard media (corn meal, agar, sugar, yeast, and 4-hydroxybenzoate) at 22°C at constant humidity on a 12:12 light/dark cycle. Fly lines were obtained from Bloomington Drosophila Stock Center (BDSC; see Table 1 for a full list of all fly stocks used).

**Table 1.** Fly lines, genotypes, abbreviations, and BDSC stock numbers used in the current study

Genotype	Abbreviation	BDSC stock #
OK6-Gal4	OK6-Gal4	64199
UAS-dilp2-GFP	dilp2-GFP	**–**
UAS-khc-RNAi	khc-RNAi	35770
UAS-unc-104-RNAi	unc-104-RNAi	58191
UAS-Cana-RNAi	Cana-RNAi	42640
UAS-Cmet-RNAi	Cmet-RNAi	35816
UAS-khc-73-RNAi	khc-73-RNAi	36733
UAS-Kif3c-RNAi	Kif3c-RNAi	40886
UAS-khc-RNAi	khc-RNAi	36795
UAS-Klp3A-RNAi	Klp3A-RNAi	40944
UAS-Pav-RNAi	Pav-RNAi	42573

### Dissection

Wandering stage third-instar larvae were picked from the walls of the vials and dissected at room temperature in calcium-free hemolymph like HL3 saline (Feng et al., 2004). The saline consists of the following (in mM): 70 NaCl, 5 KCl, 20 MgCl_2_, 10 NaHCO_3_, 115 sucrose, 5 trehalose, and 5 HEPES, pH 7.18–7.19. Dissection was conducted as previously reported (Ormerod et al., 2013); briefly, the larvae were placed dorsal side up and pinned in the anterior and posterior ends with minutien pins. Using fine scissors, a mid-dorsal cut was made along the length of the animal. The dissected tissue was stretched and pinned down, and any viscera were removed using super-fine forceps, leaving the brain and segmental nerve branches (SNBs) intact.

### Fecundity assay

Motor neuron (MN)-selective knockdown of motor proteins was conducted using RNA interference (RNAi) lines obtained from BDSC (Table 1). UAS-RNAi stock lines for genes of interest were crossed with the MN-specific Gal4-driver, OK6-Gal4. On the day of their eclosion, three males and three virgin females were placed in a vial for 3 d and then transferred into collection cages placed on top of 35 mm Petri dish with grape agar and yeast paste. The grape agar was made with 0.25 ml of grape juice, 0.75 ml of water, 0.03 g of agar, and 0.003 g of sucrose. Active dry yeast and DI water were mixed to make the yeast paste. Flies were left in the collecting cages for 26 h, and afterward the flies were removed, and the numbers of eggs were recorded. The eggs were subsequently transferred to a new Petri dish with yeast paste and left for 24 h. The number of hatched eggs was recorded, and larvae were carefully transferred into a clean food vial. The number of those larvae which matured to third-instar, pupariation, and posteclosion were tabulated.

### DCV trafficking

Wandering third-instar larvae of each genotype investigated were dissected and filleted, pinned into Sylgard bottomed 35 mm Petri dishes in standard HL3.1 saline. Live imaging was conducted using either a Nikon Eclipse microscope, equipped with a Lumencor fluorescence light engine and a Hamamatsu Orca-fusion camera, or a Zeiss Axio Imager 2 microscope with a spinning-disc confocal head (CSU-X1, Yokagawa) and ImagEM X2 EM-CCD camera (Hammamatsu). Live images were acquired using a water immersion objective, Olympus LUMFL N 60× with a 1.10 NA at either 4 Hz (confocal) or 10 Hz (fluorescence). Videos were exported from either the Nikon Elements software or Volocity 3D Image Analysis software (PerkinElmer). Time-series images were imported into Fiji and processed using the Kymograph Clear plugin. DCV time-series recordings were further processed using Kymograph Direct to compute velocity measurements. Velocity was calculated from a minimum of 50 DCVs per animal, and the average velocity of DCVs was plotted for 20 different animals. DCV flux was calculated by drawing a line perpendicular to the axon and quantifying the total number of anterograde and retrograde DCVs moving over the line per unit time. To examine the number of DCVs captured at synaptic terminals, live imaging experiments were conducted on semi-intact animals with the CNS intact. Capture was quantified as DCV entering a synaptic bouton and dropping below the focal plane.

### NMJ and muscle immunohistochemical staining

Immunohistochemistry was performed as described previously (Michael et al., 2024). Briefly, dissected third-instar larvae were fixed for 10 or 3 min in 4% paraformaldehyde (PFA) for NMJ and muscle staining, respectively. The larvae were washed three times for 10 min per wash cycle using 1× phosphate-buffered saline with 0.05% Triton X-100 (PBST). The larvae were incubated in primary antibody in PBST at room temperature for 2 h and washed in PBST three times for 10 min. Then larvae were incubated in secondary antibody in PBST at room temperature for 2 h and washed in PBS three times for 10 min. The antibodies employed in this study are as follows: mouse anti-bruchpilot (Developmental Studies Hybridoma Bank, stock #nc82, 1:500), phalloidin conjugated with Alexa Fluor 488 (Thermo Fisher Scientific, catalog #A12379, 1:4,000), anti-horseradish peroxidase (anti-HRP, Jackson ImmunoResearch Laboratories, catalog #123-605-021, 1:500), and goat anti-mouse Alexa Fluor 488 (Thermo Fisher Scientific, catalog #A11001, 1:1,000). The larvae were mounted on standard microscope slides using glycerol, and a coverslip was placed on top. The larvae were imaged using the Nikon scope described in DCV trafficking above using 60× water immersion or 60× oil immersion objective lenses. Image analysis was performed using the Nikon Elements software.

### Electrophysiology

Larvae were dissected as outlined above in a modified HL3.0 saline with the following composition (in mM): 70 NaCl, 5 KCl, 1 CaCl_2_, 4 MgCl_2_, 10 NaHCO_3_, 5 trehalose, 115 sucrose, and 5 HEPES, pH was adjusted to 7.18–7.19, daily. Minutien pins were used to pin larvae dorsal side up and the CNS was excised. Motor nerve branches were stimulated (AMPI Master 8) using a suction electrode at 0.2 Hz. Intracellular voltage of muscle fiber 6 in abdominal segments A2-A4 was recorded using sharp recording electrodes (40–60 MΩ) filled with 3 M KCl. Voltage traces were digitized using a 1550 Digitizer (Molecular Devices) and visualized using the AxoScope (Molecular Devices) software. Only recordings with a resting membrane potential of −55 to −70 mV were considered for analysis. EJPs and miniature excitatory postsynaptic potentials (minis) were quantified using ClampFit, and data were exported to Microsoft Excel for data compilation and subsequently to GraphPad prism for statistical analysis and figure making.

### Force transducer assay

Force transducer setup has been described in detail previously (Ormerod et al., 2022). Briefly, force recordings were obtained using the Aurora Scientific 403A force transducer system (Aurora Scientific), including the force transducer headstage, amplifier, and digitizer. A Master 8 (AMPI) stimulator was used to generate nerve-evoked contractions. The duration of single impulses was 5 × 10−4 s and the interburst duration was kept constant at 15 s. Larvae were dissected as outlined above. To attach larvae to the force transducer, a hook was made from a fine minutien pin and placed onto the posterior end of the larvae. For each animal, six replicate contractions were elicited for each animal sequentially, from the following stimulation frequencies: 1, 5, 10, 15, 20, 25, 30, 40, 50 100, and 150 Hz. The duration of the burst was kept constant at 600 ms. Digitized data were acquired using the Aurora Scientific software, Dynamic Muscle Acquisition Software (DMCv5.5). The digitized data were imported and processed in MATLAB using custom code (Ormerod et al., 2022).

### Larval crawling assay

Third-instar larvae were isolated from population vials, washed seven times in deionized water, and groups of 5–10 larvae from each genotype were placed in the center of a 1% agar plate for a 5 min crawling session. Seven recordings per genotype were captured using an infrared camera in a 24″ cubed black box and saved in uncompressed AVI format. The videos were converted to UFMF (Micro Fly Movie Format) using any2ufmf.exe, and CTRAX software extracted CSV files containing *X* and *Y* coordinates (in pixels) and heading (in radians). Custom Python and Excel scripts were then used to calculate distance per frame, displacement, instantaneous velocity (using central differences), and linearity (defined as net displacement divided by total displacement over 30 s segments). To account for noise and sudden leaps in position, a Hardle–Steiger sliding-window quantile method was employed for robust smoothing, and outliers were identified both manually and programmatically based on domain-specific criteria. Tracks were segmented by detecting frame gaps and tracking errors, with edge frames trimmed, and mean parameters for each 30 s segment were calculated to reveal genotype-specific locomotor traits.

### Statistical analysis

Analysis was conducted using the GraphPad Prism software. For each dataset, the relevant statistical tests were applied, and the results, including *F* and *p* values, are presented in the results section. Comparisons were made to control groups unless otherwise specified. Sample sizes were determined based on normality testing. Data are shown as the mean ± SEM (*p < 0.05; **p < 0.01; ***p < 0.001; n.s., not significant).

## Results

In *Drosophila*, there are at least 10 kinesin families: kinesin 1 (kinesin heavy chain and kinesin light chain), kinesin 2 (Kif3C, Klp64D, Klp68D), kinesin 3 (unc-104, Klp98Aand KHC-73), kinesin 4 (Klp3A and Kif21A), kinesin 5 (Klp61F), kinesin 6 (Subito and Pavarotti), kinesin 7 (cmet and cana), kinesin 8 (Klp67A), kinesin 12 (Klp54D), kinesin 13 (Klp10A,Klp59C, Klp59D), and kinesin 14 (nonclaret disjunctional; Goldstein and Gunawardena, 2000). Kinesins play key roles in several cellular processes such as chromosomal segregation during mitosis, chromosome positioning, microtubule depolymerization, and intracellular trafficking of SVs, NPs, mitochondria, and organelles (Hall and Hedgecock, 1991; Sawin et al., 1992; Yen et al., 1992; Tokai et al., 1996; Saito et al., 1997). Two families of kinesins, 1 and 3, have been identified and shown to be critical in regulating the trafficking of organelles like SVs, NPs, and mitochondria, and some evidence exists for kinesins 2 and 4 in neuronal trafficking (Hall and Hedgecock, 1991; Okada et al., 1995; Hirokawa, 1998; Glater et al., 2006; Pilling et al., 2006; Hirokawa et al., 2009; Kern et al., 2013; Liao et al., 2018).

We conducted a targeted genetic screen using all available RNAi lines against kinesins from the Blooming Drosophila Stock Center. We were able to acquire RNAi lines to knock down nine different kinesin proteins including six different families of kinesins: kinesin 1 (kinesin heavy chain, kinesin light chain), kinesin 2 (Kif3C), kinesin 3 (unc-104, khc-73), kinesin 4 (Klp3A), kinesin 6 (pav), and kinesin 7 (cmet and cana). Kinesin 1 and 3 have been shown to be critically involved in vesicle transport, including in the transport of the NPs islet cell diabetes autoantigen-1 in *C. elegans* and atrial natriuretic factor (ANF) in *Drosophila* (Pilling et al., 2006; Kern et al., 2013; Lim et al., 2017; Liao et al., 2018; Gavrilova et al., 2024). Kif3C has been demonstrated as a microtubule-destabilizing factor required for axon regeneration rather than cargo transport (Gumy et al., 2013). Cana and cmet, *Drosophila* homolog of CENP-E, have been shown to play key roles during stages of cell division, mediating processes such as chromosome alignment, spindle formation, and kinetochore assembly (Yen et al., 1992; Maia et al., 2007; Demirdizen et al., 2019). In mice, KLP3A mutations are understood to disrupt central spindle and cytokinesis in males, although somatic cells in *Drosophila* show insensitivity to loss of wild-type KLP3A (Williams et al., n.d.). On the other hand, KHC-73 has been shown to facilitate the routing of synaptic endosomes back to the soma (Liao et al., 2018). Finally, Pavarotti forms the centralspindlin complex, which plays a role during cytokinesis, but evidence for a role in neurons via interactions with kin-1 to regulate microtubule sliding and neurite outgrowth is lacking (Adams et al., 1998; Del Castillo et al., 2015).

### Targeted genetic screen for motors involved in NP sorting and trafficking

**Figure 1.**
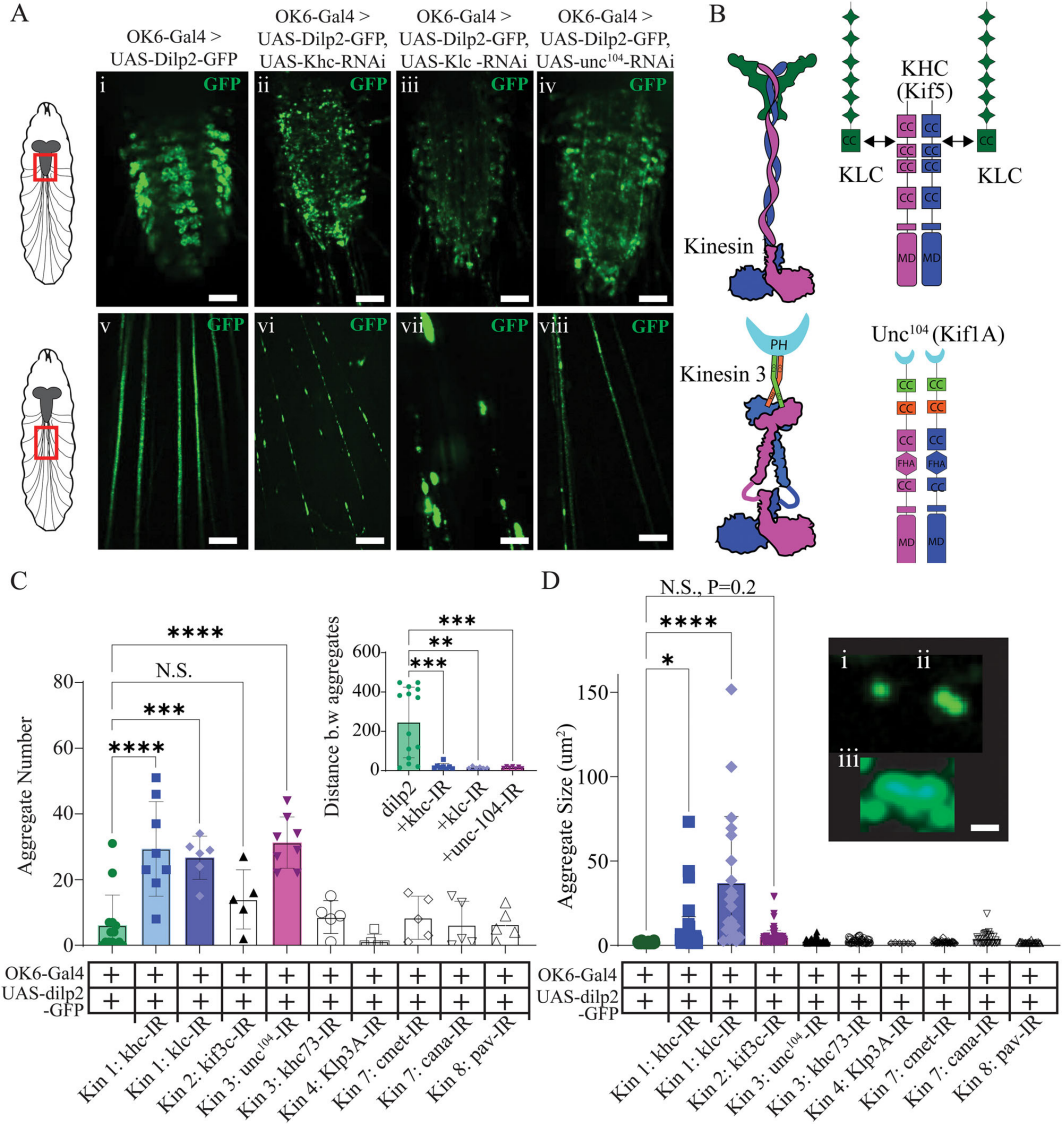
Kinesin disruption critically alters NP trafficking in the nervous system. ***A***, Confocal imaging from the VNC, and SNBs, highlighted in diagram *Drosophila* third-instar larvae using a red box on left inset, top, and bottom, respectively. ***Ai, v***, from control (OK6-Gal4 >dilp2-GFP); ***Aii, vi***, kinesin 1 khc knockdown (OK6-Gal4>dilp2-GFP, khc-RNAi); ***Aiii, vii***, kinesin 1 khc knockdown (OK6-Gal4>dilp2-GFP, klc-RNAi); and ***Aiv, viii***, kinesin 3 unc-104 **(**OK6-Gal4>dilp2-GFP, unc-104-RNAi). Scale bar, 50 microns. ***B***, Schematic of kinesin 1 (top) and kinesin 3 (bottom), highlighting the putative structural organization (left) and functional domains right (cc, coiled coil; md, motor domain; PH, Pleckstrin homology; fha, forkhead-associated). ***C***, Quantification of the total number of aggregates from six kinesin families and nine different kinesin-related proteins. Table below indicates the presence of OK6-Gal4, UAS-dilp2-GFP, and the inverted repeat (IR) for the kinesin. Inset, Average distance between aggregates for the four genotypes shown units, microns. ***D***, Average aggregate size for the 9 klps investigated. The table below indicates the presence of OK6-Gal4, UAS-dilp2-GFP, and the inverted repeat (IR) for the kinesin. Inset, Representative image of a (***i***) single, (***ii***) double, and (***iii***) nonmotile DCV aggregate.

To examine the impact of knocking down kinesin motor proteins on NP trafficking in MNs, fluorescently tagged *Drosophila* insulin-like peptide 2 (UAS-dilp2-GFP; dilp2-GFP) was expressed in MNs using the OK6-Gal4 driver (Sanyal, 2009; Wong et al., 2012). Flies expressing dilp2-GFP showed a stereotyped expression pattern within the ventral nerve cord (VNC) and SNBs projecting out to body-wall muscles (Fig. 1*A*). Next, OK6-Gal4>dilp2-GFP was crossed with nine RNAi lines for candidate kinesin-like peptides (klps), and subsequently, NP aggregation in the VNC and SNBs was examined (Fig. 1). Of those RNAi lines examined, only three lines, khc, klc, and unc-104, revealed a significant increase in the number of NP aggregation events observed in SNBs. Aggregation was defined as any nonmobile fluorescent puncta, which exceeded 1.25 µm^2^ (Fig. 1*C*, one-way ANVOA, *F* = 11.8; *p* < 0.0001; *N* = 5-8). A quantification of the number of aggregates revealed a ∼5-fold increase in NP-aggregate number compared with controls when knocking down unc^104^, khc, or khc (5.1, 4.8, 4.3, respectively; one-way ANOVA, *F* = 32.4; *p* < 0.0001; *N* = 5–8). The inset in Figure 1*C* shows the average distance between aggregates in microns for control and knockdown of khc, klc, and unc-104 (one-way ANOVA, *F* = 10.7; *p* = 0.0001; *N* = 10–14). Thus, the aggregation in knockdown lines is more plentiful and clustered. Next, the aggregate size was tabulated revealing a significant increase in the size of fluorescent aggregates compared with controls for knockdown of khc and klc (Fig. 1*D*, one-way ANOVA, *F* = 11.8; *p* < 0.0001). No significant increase in the aggregate size was observed for unc-104 or any of the other RNAi lines from kinesin 2, 4, 6, or 7. Taken together, only knocking down members of kinesin families 1 and 3 led to significant NP aggregation in the VNC and SNBs of motor axons.

### Kinesin knockdown impairs trafficking of NPs

**Figure 2.**
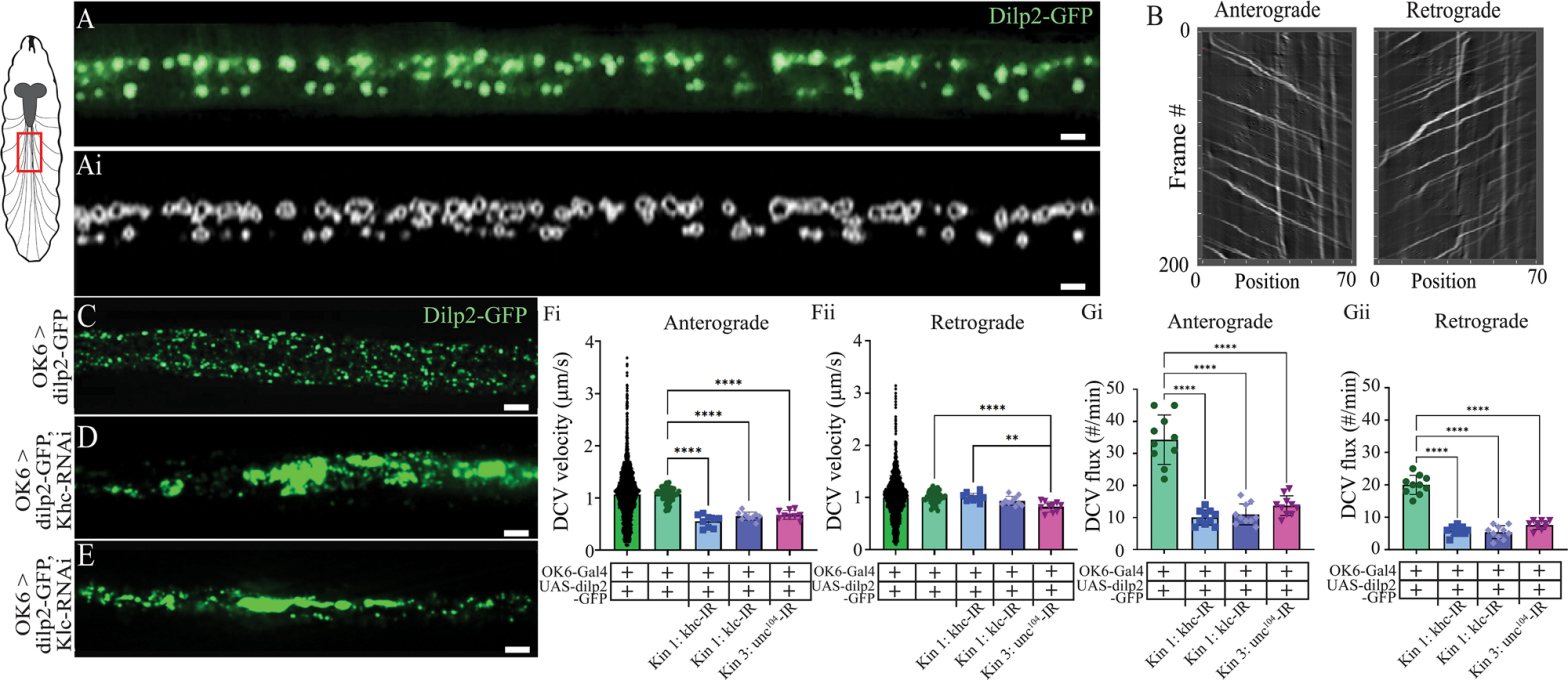
Axonal trafficking of NPs is significantly altered in several kinesin protein knockdowns. Left, Schematic of larvae indicating location of imaging. ***A***, SNB showing trafficking of the fluorescently tagged NP, Dilp2-GFP. ***Ai***, Same axonal tract as ***A*** after computational tracking and identification of individual DCVs. Scale bar, 4 microns. ***B***, Kymographs created from the software Kymograph Direct. Representative SNB images from (***C***) OK6>Dilp2-GFP, (***D***) OK6>Dilp2-GFP, khc-RNAi, (***E***) OK6>Dilp2-GFP, khc-RNAi**.** Scale bar, 9 microns. Quantification of DCV velocity from within one SNB, divided into anterograde (***Fi***) and retrograde (***Fii***). Control data, OK6-dilp2-GFP is demonstrated in two bars. Left showing 1,000 data points, 50 DCV/animal for 20 animals; right, the 50 DCVs per animal averaged for each animal and plotted for the remaining genotypes. ***G***, Quantification of DCV flux for the four genotypes separated into (***Gi***) anterograde and (***Gii***) retrograde.

To examine the impacts of kinesin 1 and 3 knockdown on molecular trafficking of NPs, live imaging of NP trafficking in SNBs was conducted using a spinning-disc confocal microscope (Fig. 2). Estimates of DCV velocity were initially measured manually; then subsequently, a computer software (ilastik) was used to isolate individual DCVs and then track their position from frame to frame (Fig. 2*A*; Movies 1 and 2). This software exports position data for each frame, for each DCV, from which velocity (microns/second) and flux (the number of DCVs moving past a fixed position along the axon per minute) can be extracted. Kymographs were also created using Kymograph Clear and Kymograph Direct (Fig. 2*B*). Live trafficking of OK6>dilp2-GFP in SNBs reveals abundant and coordinated antero- and retrograde trafficking (Fig. 2*C*; Movies 1 and 2). However, live imaging from khc, klc, and unc^104^-RNAi knockdown lines shows impacts of motor protein knockdown of NP trafficking (Fig. 2*D*,*E*). To quantify DCV velocity from OK6>dilp2-GFP controls, we recorded one SNB from 20 different third-instar larvae. Fifty individual DCVs were measured per SNB, and data were separated into two pools, anterograde and retrograde directions, and all 1,000 data points were plotted to show the spread of all data (Fig. 2*Fi*,*ii*, left bars). For the remaining bars, data from 50 DCVs were averaged from each SNB from one animal and plotted as a single data point. No significant difference was observed in the rate of DCV trafficking between anterograde and retrograde trafficking in OK6>dilp2-GFP (anterograde, 1.07 ± 0.11 microns/s; retrograde, 1.00 ± 0.11 microns/s; *T* test, *F* = 1.2; *p* = 0.2; *N* = 20; 50 DCVs/animal). Knocking down khc, klc, and unc^104^ all significantly reduced the velocity of anterograde DCV trafficking (0.55, 0.65, and 0.68 microns/s, corresponding to 48, 39, and 36% reduction, respectively). However, knocking down khc and klc did not significantly impair retrograde trafficking, while surprisingly, knocking down unc^104^ significantly reduce the velocity retrograde DCV trafficking, indicating a potential role for kinesin 3 in retrograde trafficking (Fig. 2*Fii*).

**Movie 1.** Real-time, in vivo intracellular trafficking of fluorescently tagged NPs (OK6-Gal4>UAS-dilp2-GFP). [View online]

**Movie 2.** Postprocessed video using computer software (ilastik) to isolate individual DCVs and then track their position from frame to frame. [View online]

Given the prevalence of aggregates in the kinesin 1 and 3 knockdown lines, the bulk transport of material moving through axons should be reduced. Indeed, the total number of DCVs moving both retrograde and anterograde was significantly reduced when knocking down either hc, klc, or unc^104^ (Fig. 2*Gi*, anterograde, one-way ANOVA, *F* = 61.5; *p* < 0.0001; *Gii*, anterograde, one-way ANOVA, *F* = 57.1; *p* < 0.0001; *N* = 8–10). While previous investigations have demonstrated a role for these motors in axonal trafficking, there are sparse data exploring the downstream impacts of impaired axonal trafficking on structure, function, physiology, and behavior of the animal.

### Kinesin motor protein knockdown severely impacts MN gross and ultrastructural morphology at the NMJ but not muscles

**Figure 3.**
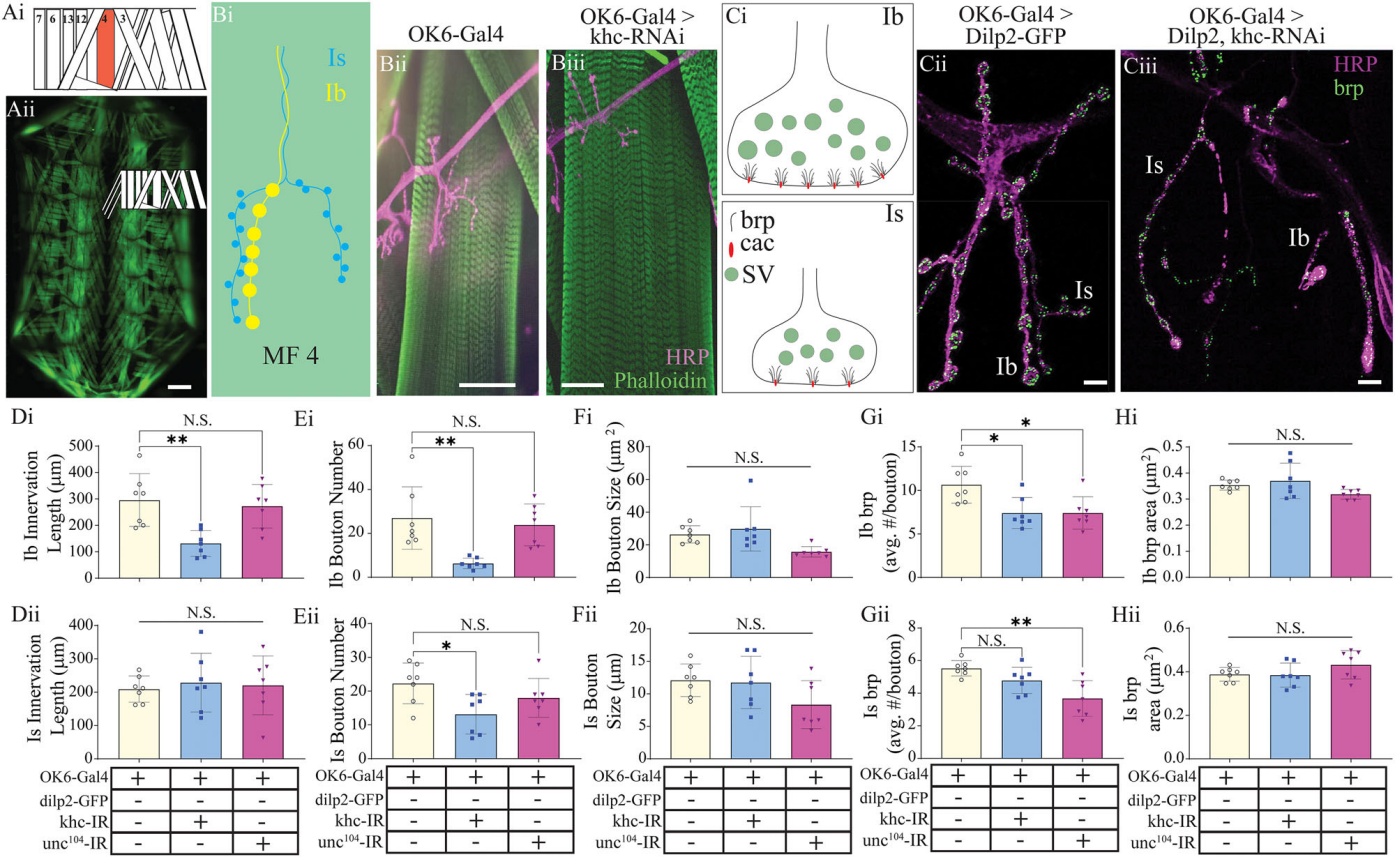
Targeted genetic knockdown of kinesins 1 and 3 significantly impair NMJ formation. ***Ai***, Schematic depiction of third-instar body-wall muscles within a single abdominal hemisegment. ***Aii***, Fluorescence image of dissected third-instar larvae, highlighting a single abdominal hemisegment in ***Ai***. Scale bar, 500 microns. ***Bi***, Schematic depiction of glutamatergic Ib and Is innervation along the surface of MF 4. ***Bii***, Immunostain from OK6-Gal4 and (***Biii***) OK6-Gal4>UAS-khc-RNAi: with HRP, purple; phalloidin, green. Scale bar, 50 microns. ***Ci***, Schematic glutamatergic Ib (top) and Is (bottom) boutons depicting bruchpilot (brp), cacophony (cac), and SV. ***Cii***, Immunostain from OK6-Gal4>UAS-dilp2-GFP, green, and (***Ciii***) OK6-Gal4>UAS-khc-RNAi, with HRP, purple, and DCV, green. Scale bar, 4 microns. Quantification of changes in innervation length (***D***), bouton number (***E***), bouton size (***F***), brp density (average number per bouton, ***G***), and average BRP area (***H***), separated by Ib (***i***, top) and Is (***ii***, bottom) MN subtypes.

**Figure 4.**
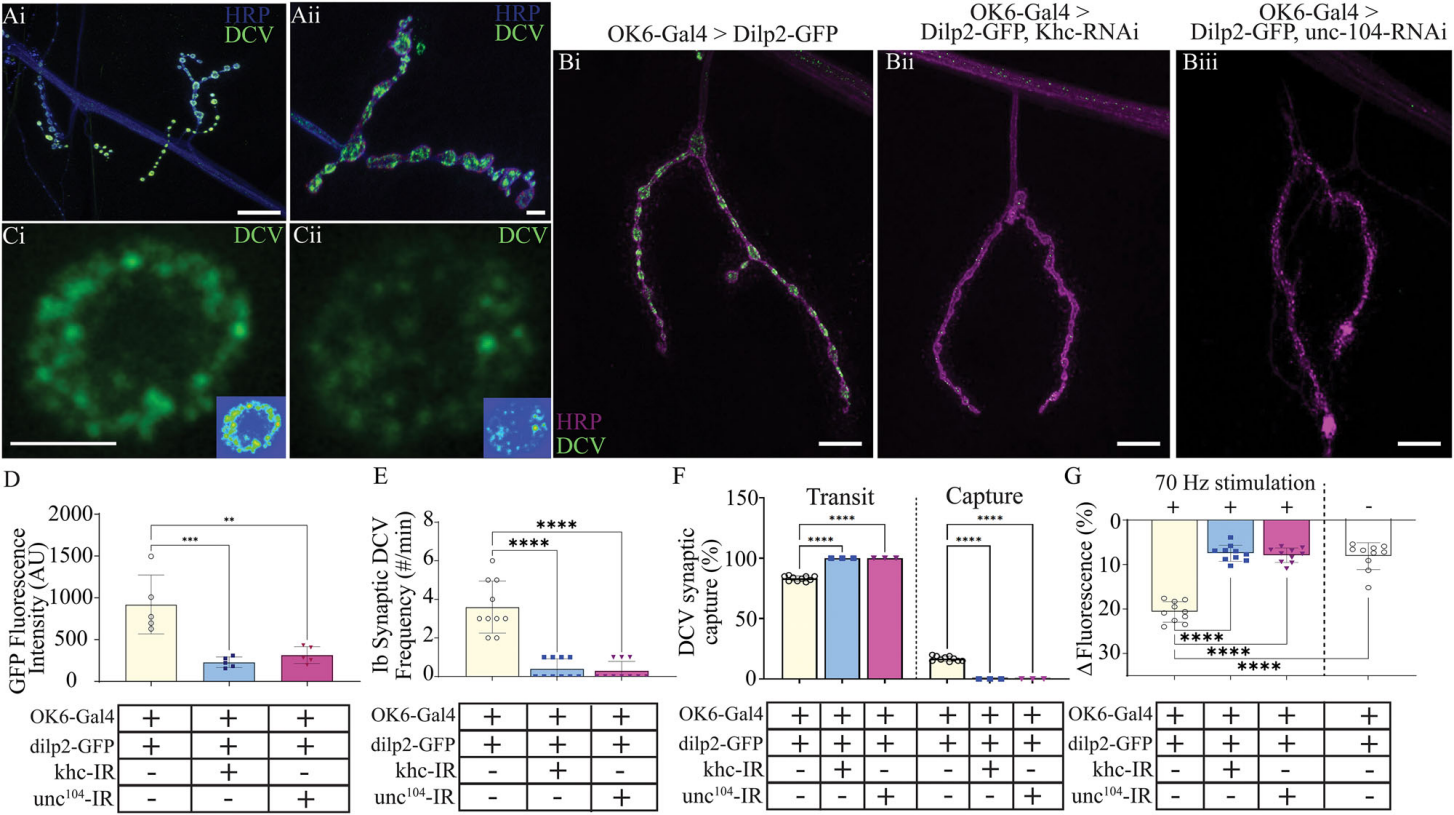
Muscle ultrastructure is not altered in kinesin 1 or 3 knockdown lines. Immunostains from dissected larvae showing body-wall muscles in a single abdominal hemisegment from (***Ai***) OK6-Gal4 and (***Aii***) OK6-Gal4>UAS-khc-RNAi. ***Ai’*** and ***Aii’*** depict confocal images highlighting MF 4 from OK6-Gal4 and OK6-Gal4>UAS-khc-RNAi, respectively. The arrow indicates location and direction of fluorescence intensity profile line. ***B***, Fluorescence intensity profile line for GFP (phalloidin). Dashed white and magenta lines indicate how sarcomere and I-band measurements were calculated, respectively. Quantification of muscle length (***C***), muscle width (***D***), muscle area (***E***), sarcomere length (***F***), and I-band (***G***).

The larval SNBs projecting from the VNC innervate supercontractile body-wall muscles in the periphery responsible for larval movement, like peristaltic locomotion (Ormerod et al., 2022). There are four different MN subtypes which innervate body-wall muscles including the two glutamatergic subtypes Ib and Is, as well as the neuromodulatory type IIs, and lastly the insulin-like immunoreactive type III (Peron et al., 2009). The type Ib and Is (comparable to mammalian tonic and phasic, respectively) propagate the output from the CNS underlying rhythmic muscle contractions (Ormerod et al., 2022). To assess changes in NMJ morphology and physiology, we first examined the innervation of Ib and Is MNs innervating muscle fiber 4 (MF 4; Fig. 3). An assessment of the impact of kinesin knockdowns on MF 4 NMJ was initially conducted by quantifying the number of boutons, size of boutons, and innervation length of the NMJ along the surface of the muscle (Fig. 3*B*,*E*). While there were no significant effects on the morphology of NMJ innervation of MN-Is, the extent of innervation by MN-Ib was significantly reduced for khc knockdown animals (Fig. 3*Di*, one-way ANOVA, *F* = 8.6; *p* < 0.01). Interestingly, the number of MN-Ib and MN-Is boutons innervating MF 4 was significantly reduced for khc mutants (Fig. 3*Ei*, one-way ANOVA, *F* = 8.7; *p* < 0.01; *Eii*, *F* = 4.2; *p* < 0.05). No change in the bouton size was observed for either MN subtype (Fig. 3*F*). To further assess changes in NMJ ultrastructure, we conducted immunostaining of bruchpilot (brp), a presynaptic active zone (AZ) scaffolding protein homologous to mammalian ELKS (Akbergenova et al., 2018). Both khc and unc^104^ mutants displayed a reduction in brp-positive puncta in MN-Ib boutons (Fig. 3, one-way ANOVA, *F* = 6.7; *p* < 0.01). However, only unc^104^ knockdown animals displayed a reduction in brp-positive puncta in MN-Is boutons, while khc knockdown animals bordered on significance with *p* = 0.056 (Fig. 3, one-way ANOVA, *F* = 8.8; *p* < 0.01; *N* = 7). Given the substantive changes in MN morphology and the intimate and dynamic relationship occurring between MNs and body-wall muscles during embryonic development, we next assessed whether knocking down motor proteins in motor axons impacted muscle morphology (Fig. 4). First, an assessment in gross muscle morphology was conducted by examining changes in individual muscle fiber length, width, and area, but no significant differences were observed (Fig. 4, one-way ANOVAs, length, *F* = 0.7; *p* = 0.5; width, *F* = 0.4; *p* = 0.7; area, *F* = 0.7; *p* = 0.5; *N* = 8, 6 muscles per animal). Next, a fluorescence line profile was conducted using phalloidin-stained muscles to approximate the length of sarcomeres and I-bands, but no significant differences in muscle morphology were observed ##(Fig. 4, one-way ANOVA; sarcomere, *F* = 0.4; *p* = 0.7; *N* = 8; I-band, *F*, *p* = 0.7; *N* = 8; for Fi, Gi, each *N* is an average of six muscles, 10–16 sarcomeres or I-bands per muscle).

### Knocking down kinesins 1 and 3 dramatically reduces NP abundance, movement, and release at the NMJ

**Figure 5.**
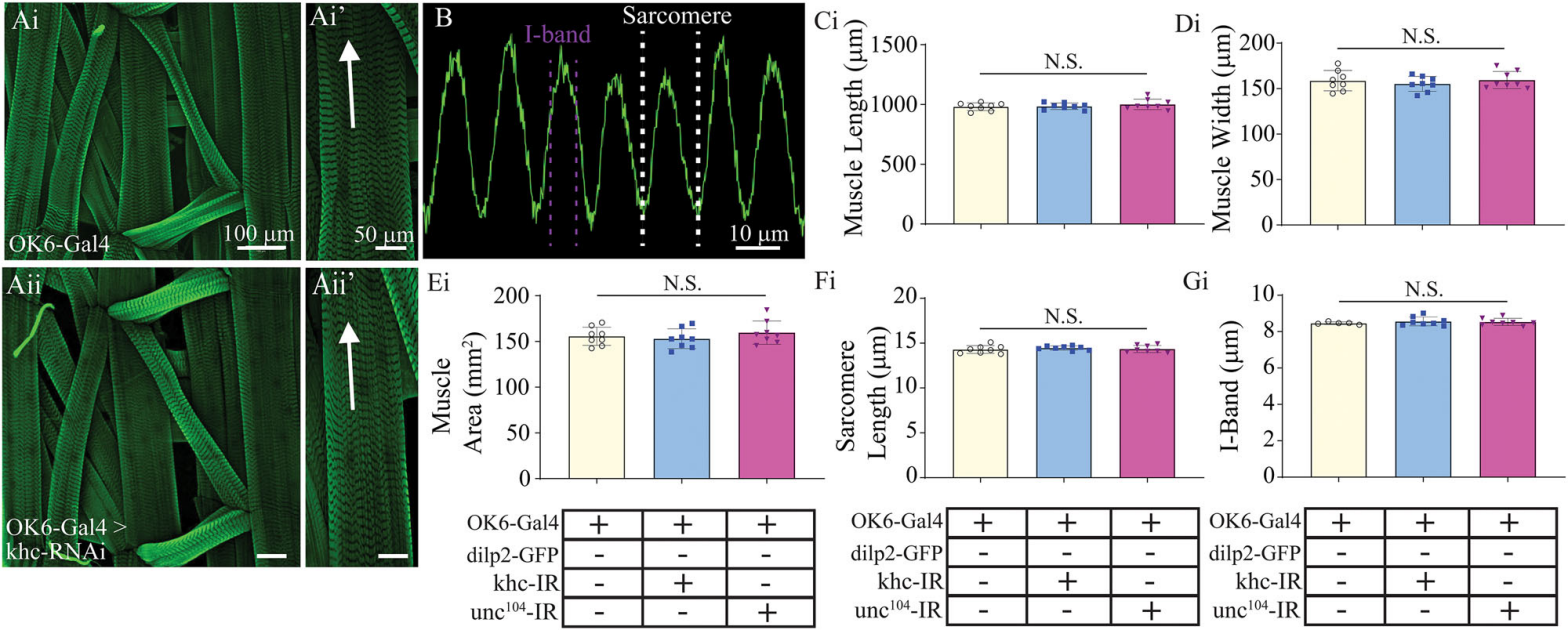
Motor protein knockdown profoundly impacts DCV mobility and release at synaptic boutons. Immunostained images showing (***Ai***) peripheral nerve branches and multiple strings of synaptic varicosities and (***Ai’***) zoomed-in image of a single string of synaptic varicosities from OK6-Gal4>dilp2-GFP, stained with HRP, blue, and DCVs, green. A representative image from a single bouton taken during live spinning-disc confocal imaging before (***Bi***) and after (***Bii***) high frequency, 70 Hz stimulation. Insets, heatmap showing fluorescence intensity. Immunostains to show changes in DCV localization at synaptic varicosities in (***Ci***) OK6-Gal4>UAS-dilp2-GFP: (***Ci***) OK6-Gal4>dilp2-GFP, khc-RNAi, (***Ci***) OK6-Gal4>dilp2-GFP, unc-104-RNAi. Quantification of (***D***) changes in GFP fluorescence at synaptic varicosities, (***E***) frequency of DCVs at boutons, (***F***) number of DCVs transiting through “transiting,” and those entering boutons “synaptic capture” (***G***) drop of GFP fluorescence at nonterminal boutons following 70 Hz stimulation for 20 s.

During the initial screening of motor protein knockdown on NP aggregation and localization (Fig. 1), a substantial change in fluorescence localization in the periphery and at NMJs was noted. Representative confocal images from OK6-Gal4>dilp2-GFP show a stereotypical distribution of DCVs both within axons and at MN terminals (Fig. 5*Ai*,*ii*). However, knocking down khc results in a dramatic reduction in NP abundance in MNs along the surface of muscles and at synaptic varicosities (Fig. 5*B*). A costain for axonal membrane was conducted with anti-HRP to quantify average GFP fluorescence within axons and at NMJs along the surface of MF 4, and a significant reduction was observed for both khc and unc^104^ knockdown animals (Fig. 5*D*, OKD-Gal4, 920 ± 351, OK6-Gal4>dilp2-GFP; khc-RNAi, 228 ± 63, OK6-Gal4>dilp2-GFP; unc^104^-RNAi, 314 ± 101; one-way ANOVA, *F* = 15.4; *p* < 0.0001; *N* = 5, six muscles per animal). Using spinning-disc confocal microscopy, the movement of DCVs entering a nonterminal bouton was assessed. For OK6-Gal4>dilp2-GFP animals, an average of 3.6 ± 1.4 DCVs per minute, while for khc and unc^104^ animals, an average of 0.4 ± 0.5 and 0.3 ± 0.5 per minute, respectfully, were observed (Fig. 5*E*, one-way ANOVA, *F* = 45.5; *p* < 0.0001; *N* = 10). Next, to assess rates of synaptic capture, 50 DCVs were observed per bouton, and the total number that transited through versus were captured into boutons was counted and converted to a percentage (Fig. 5*F*). For OK6-Gal4>dilp2-GFP animals, 83.2 ± 2.1% of DCVs transited through the boutons. Given that so few DCVs were mobile and their velocity of trafficking was severely reduced, very few DCVs transited through boutons during the experiments. Consequently, for the 3–4 animals where DCV mobilization was observed through a bouton, none were captured resulting in significant differences compared with controls for both khc and unc^104^ knockdown animals (Fig. 5*F*, one-way ANOVA, *F* = 3417; *p* < 0.0001; *N* = 3–10). Lastly, to determine if knocking down kinesin 1 or 3 impaired DCV release from boutons, a “fusion assay” was conducted using previously published approaches (Levitan et al., 2007). Axons from MNs were stimulated at 70 Hz stimulation for 20 s, and DCV exocytosis was quantified as a reduction in GFP fluorescence at Ib boutons (Fig. 5*Ci*,*ii*). However, when directly stimulating boutons from either kinesin 1 or kinesin 3 knockdown animals, a significantly impaired reduction in stimulation-induced fluorescence change was observed (Fig. 5*G*, one-way ANOVA, *F* = 0.9; *p* < 0.05; *N* = 10). Any drop in fluorescence is likely a reflection of photobleaching given that no redistribution in DCV was observed, as was observed for controls (Fig. 5*Ei* vs *Eii*).

### Kinesin 1, but not 3, alters neuromuscular transduction

**Figure 6.**
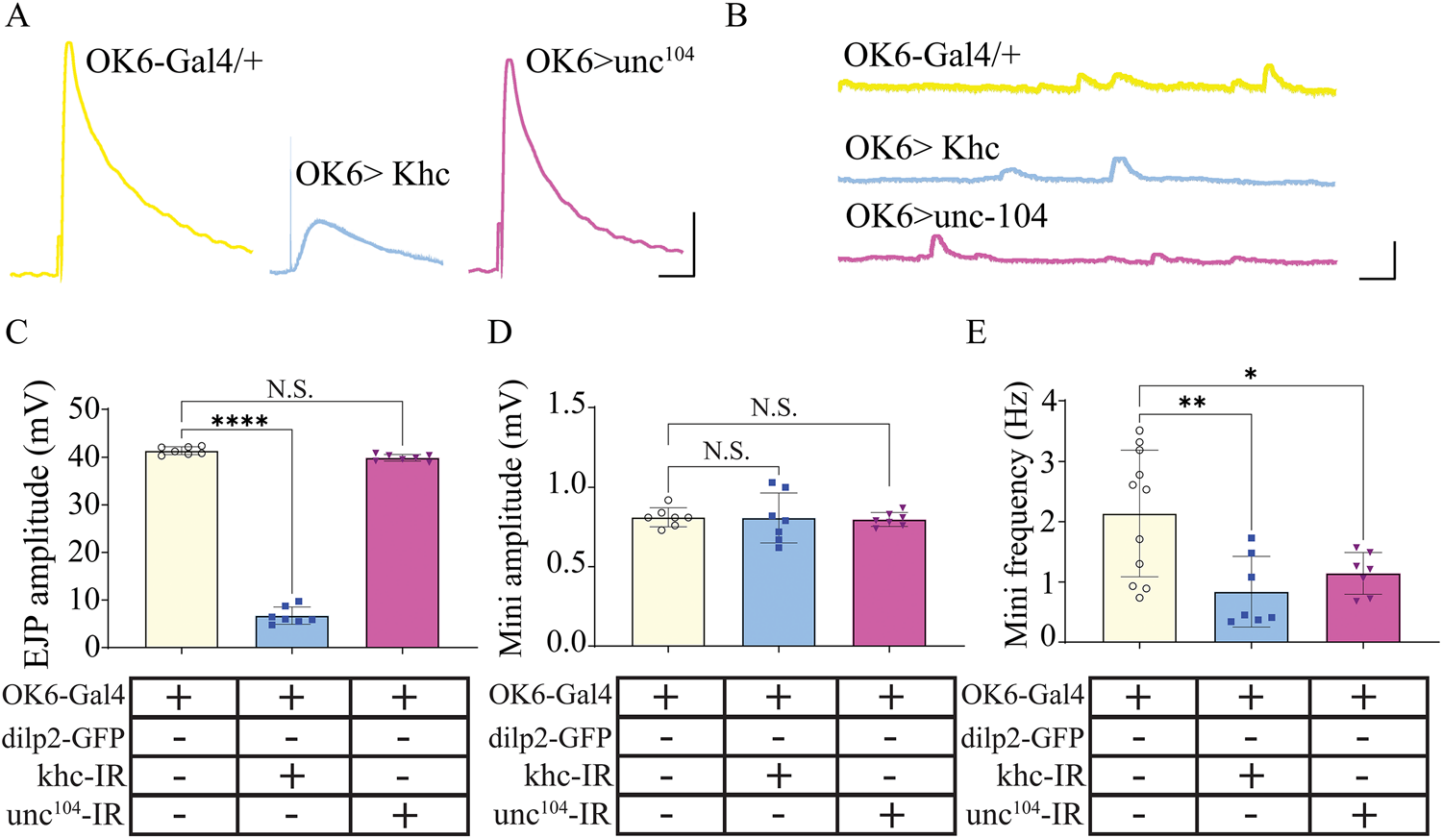
Neuromuscular transduction is significantly impaired only in kinesin 1 knockdown lines. Representative electrophysiological recordings from the three genotypes showing EJPs (***A***, EJPs) and miniature end plate potentials (***B***, minis). Quantification of changes in (***C***) EJP amplitude, (***D***) mini amplitude, and (***E***) mini frequency.

**Figure 7.**
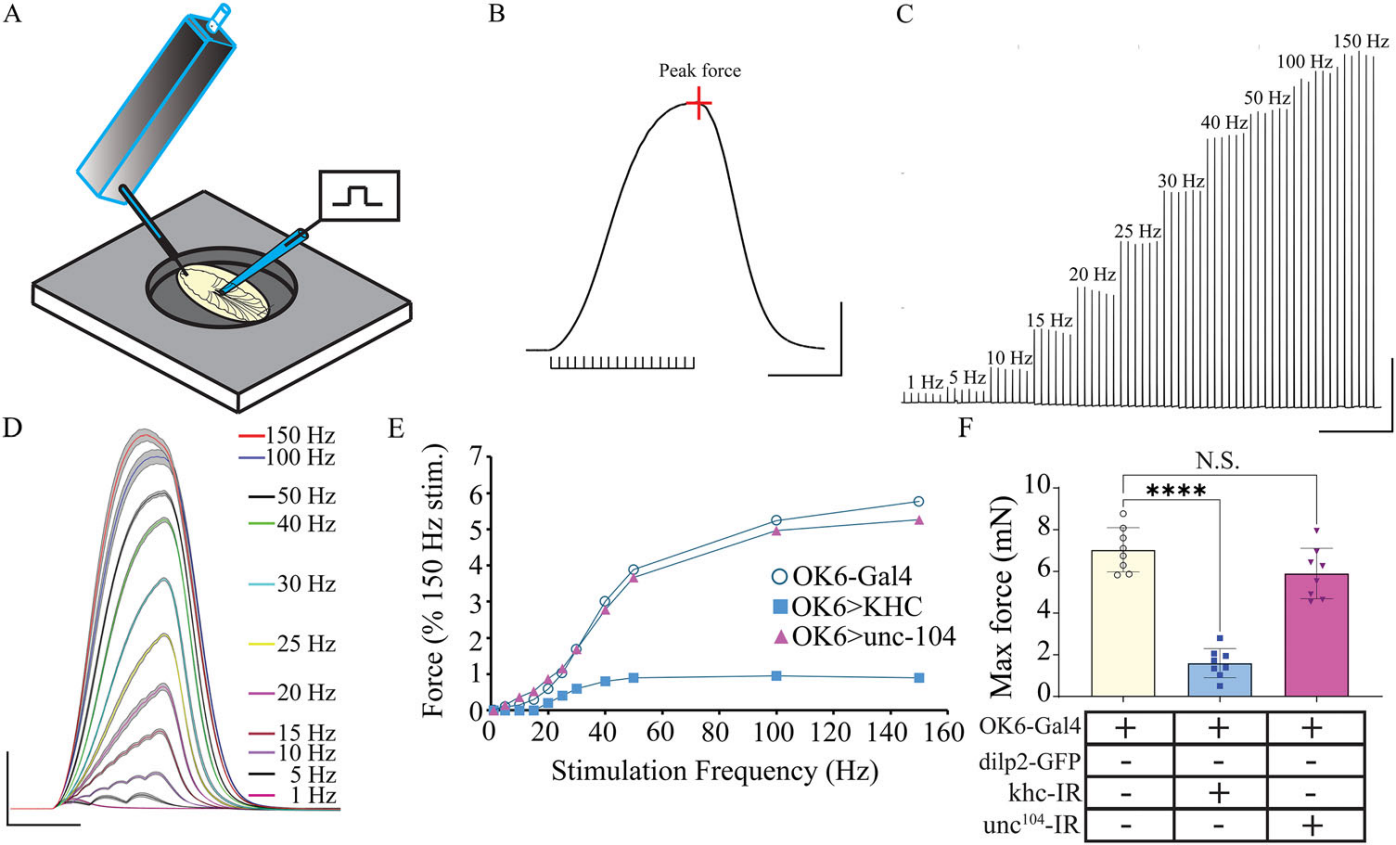
ECC is severely reduced in kinesin 1 knockdown larvae. ***A***, Schematic representation of muscle force transducer setup. ***B***, A single muscle contraction trace induced by a 40 Hz stimulation for 600 ms. ***C***, Larval motoneuron stimulation protocol used to generate dynamic force–frequency recordings for muscle contraction from initial threshold through saturation (1–150 Hz). Stimulus duration was kept constant at 600 ms. ***D***, The six replicate contractions from each stimulation frequency shown in ***C*** were averaged and plotted with 95% confidence interval (gray area). CI. ***E***, Force–frequency plot from each of the three genotypes. ***F***, Quantification of maximal force generated from 150 Hz stimulation for each of the three genotypes.

Given the dramatic changes in NMJ ultrastructure and NP mobility and release, we next assessed whether knocking down kinesin 1 or kinesin 3 impacted neuromuscular transmission (Fig. 6). These molecular motors are known to transport not only NPs via DCVs, but other cargoes like SVs, and mitochondria. Using intracellular voltage recordings from postsynaptic muscle fibers, a dramatic reduction in EJPs was observed for kinesin 1 knockdown animals, but not for kinesin 3 knockdowns compared with controls (Fig. 6*A*,*C*, one-way ANOVA, *F* = 1812; *p* < 0.0001; *N* = 7). No observable change was found for miniature EJPs (Fig. 7*B*, minis) amplitude (Fig. 6*D*, one-way ANOVA, *F* = 0.04; *p* = 0.9; *N* = 7); however, significant reduction was observed for both kinesin 1 and 3 knockdowns (Fig. 6*E*, one-way ANOVA, *F* = 6.7; *p* = 0.01; *N* = 7).

### Kinesins 1, but not 3, reduce excitation–contraction coupling

To further examine how kinesin 1 and 3 knockdown in MNs would impact the NMJ, an examination of muscle force production from larval body-wall muscle was explored. We previously established methodology to examine muscle force production by generation a force–frequency curve, spanning the entirety of the endogenous MN frequencies, ranging from 1 to 150 Hz (Ormerod et al., 2022; Fig. 7*A*–*D*). Knocking down kinesin 3 did not impair muscle force production at any point along the force–frequency curve, nor was maximal force production reduced compared with controls (Fig. 7*E*,*F*). However, knocking down kinesin 1 dramatically reduced muscle force production at every stimulation frequency investigated (Fig. 7*E*). Unlike control and kinesin 3 knockdown animals which produced muscle force from a single AP, kinesin 1 knockdown animals did not generate quantifiable force until 20 Hz stimulation was delivered (Fig. 7*E*). The reduction in force generation was >80% above 50 Hz representing one of the most dramatic phenotypes observed using this experimental approach (Ormerod et al., 2022; Hana et al., 2024; Michael et al., 2024). The maximum force generated by kinesin 3 knockdown animals was not significantly different from controls; however, a significant reduction was observed following knockdown of kinesin 1 (Fig. 7*F*, one-way ANOVA, *F* = 64.2; *p* < 0.0001; *N* = 8).

### Disruptions in kinesin 1, but not 3, result in changes in larval behavior

**Figure 8.**
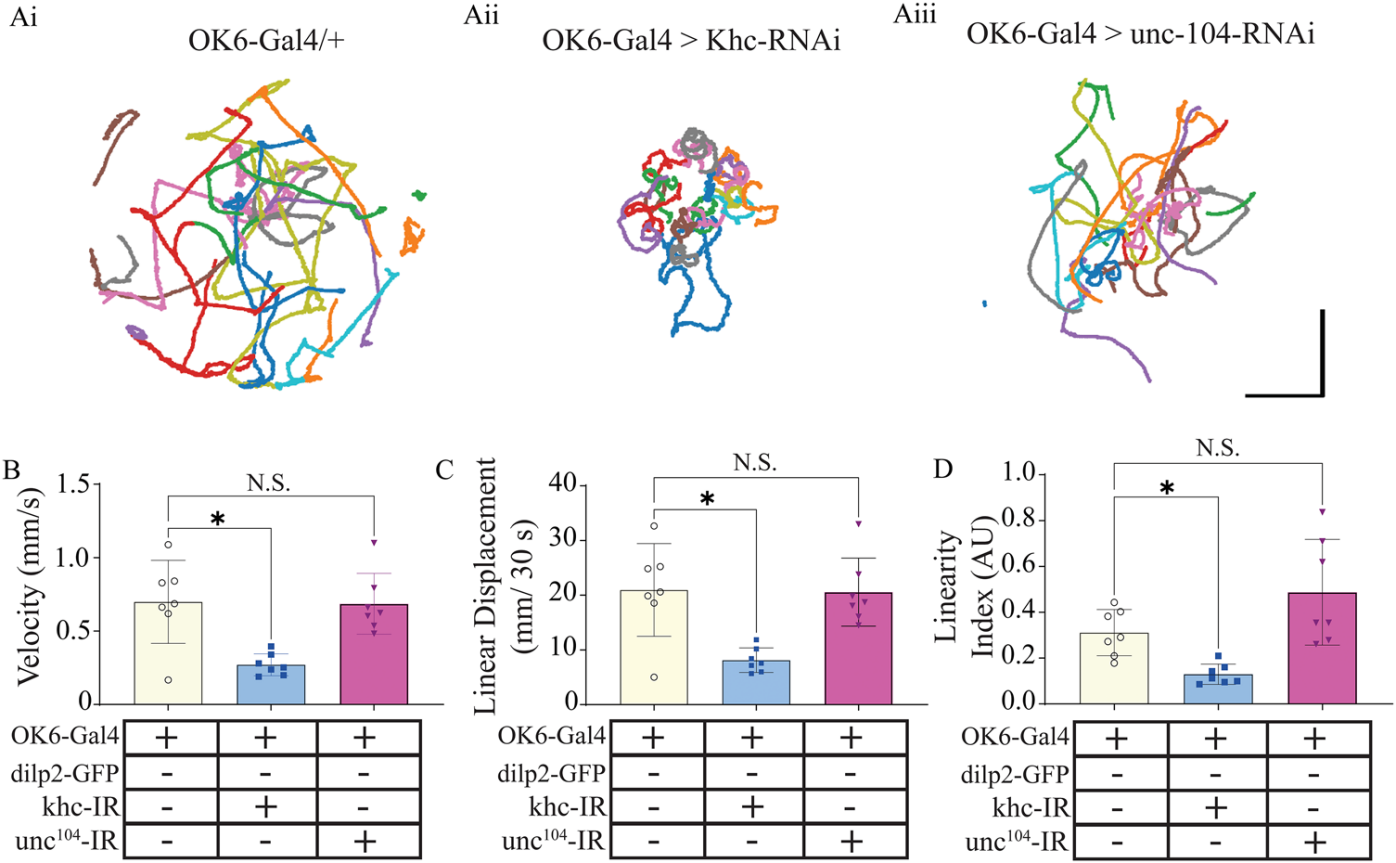
Deficits in larval crawling behavior are only observed in kinesin 1 knockdown animals. Schematic representing larval crawling behavioral changes from (***Ai***) OK6-Gal4/+; (***Ai***) OK6-Gal4>khc-RNAi, and (***Ai***) OK6-Gal4>unc-104-RNAi. ***B, C, D***, Seven videos of 10 larvae from each genotype were quantified and the positional data were averaged and plotted at a confidence interval of 95%; velocity (***B***) was measured as millimeters per second. Linear displacement (***C***) was calculated as *d* = √((*x*_end_ − *x*_start_)² + (*y*_end_ − *y*_start_)²) per 30 s segment of larval track and then averaged. Linearity index (D) was measured as the linear displacement divided by the total displacement (nonlinear), providing an index of total time spent turning versus actual distance traveled.

Lastly, to assess whole-organism behavioral changes resulting from kinesin 1 and 3 knockdowns, larval crawling was conducted. Underlying larval crawling are central pattern generators sending high-frequency patterned output from the VNC to the muscles from the CNS via MNs in the VNC 55. Consistent with electrophysiological and force recordings, kinesin 1 knockdown significantly impacted larval crawling across numerous metrics examined (Fig. 8). Ctrax was used to track larvae, and custom code was created to track quantify crawling, enabling the generation of crawling patterns (Fig. 8*Ai*–*iii*). From these, linear velocity and linear displacement (Fig. 8*B*,*C*, Brown–Forsythe ANOVA, *F* = 9.7; *p* < 0.01) and linearity index (Fig. 8*D*, Brown–Forsythe ANOVA, *F* = 10.3; *p* < 0.01) were all significantly reduced from kinesin 1 knockdown animals, but not kinesin 3 (Movies 3 and 4).

**Movie 3.** Infrared images of OK6-Gal4 third-instar larvae captured at 10 fps in the absence of ambient light. [View online]

**Movie 4.** Infrared images of OK6-Gal4<UAS-khc-RNAi third-instar larvae captured at 10 fps in the absence of ambient light. [View online]

### Kinesin 1 and 3 motor protein disruption significantly effects growth and development

**Figure 9.**
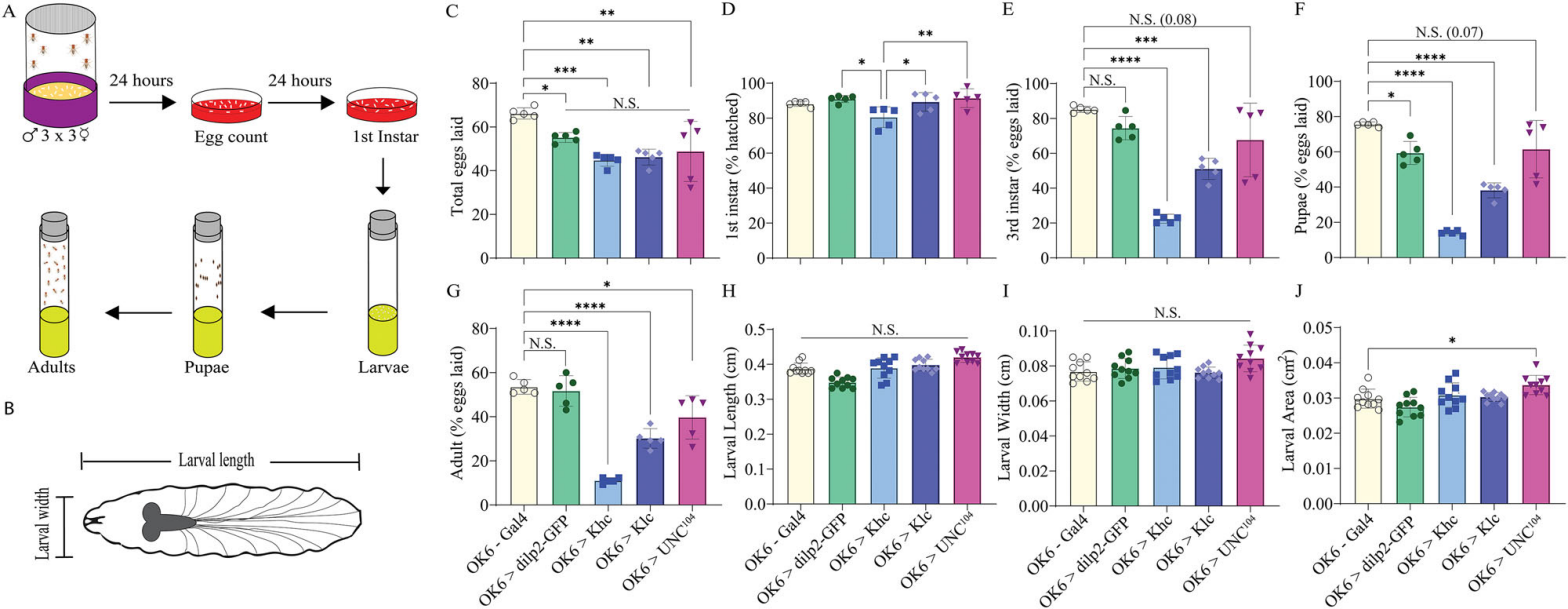
Disruptions in kinesin protein expression significantly alters fecundity but not larval morphology. ***A***, Schematic mating and egg-laying assay to track animals throughout development. ***B***, Schematic third instar with length and width measurements. For the five genotypes investigated (OK6-Gal4/+, yellow; OK6-Gal4>UAS-dilp2-GFP, green; OK6-Gal4>UAS-khc-RNAi, light blue; OK6-Gal4>UAS-khc-RNAi, dark blue; OK6-Gal4>UAS-unc-104-RNAi, pink), the total number of eggs were counted (***C***), the number of eggs that hatched to first instar (***D***), progressed to third instar (***E***), pupae (***F***), and adults (***G***). For third-instar larvae, measurements of length (***H***), width (***I***), and area (***J***) are plotted for each genotype.

First, we examined whether knocking down kinesin 1 (khc and klc) or 3 (unc-104) impacted the growth and development of the animals (Fig. 9). To do so, we mated three males and three females 72 h posteclosion for 24 h and tabulated the total number of eggs laid (Fig. 9*A*). Significant differences were observed between OK6-Gal4 control flies and flies with knockdown of khc, klc, and unc-104, but no significant differences were observed between different kinesin knockdown lines (Fig. 9*C*, one-way ANOVA, *F* = 8.7; *p* < 0.001; *N* = 5). After 24 h, the number of eggs that hatched to first instar was tabulated and larvae transferred to fresh food (Fig. 9*A*). Knocking down khc significantly reduced the viability of eggs, while khc and unc-104 knockdowns were not significantly different from controls (Fig. 9*D*, one-way ANOVA, *F* = 4.9; *p* = 0.01; *N* = 5). The number of first instars the developed to third instars was significantly reduced for both khc and khc knockdown animals compared with control; however, no significant impact was observed for unc-104 animals (Fig. 9*E*, one-way ANOVA, *F* = 27.8; *p* < 0.0001; *N* = 5), and similar results were observed for the number of larvae that developed to pupa (Fig. 9*F*, one-way ANOVA, *F* = 43.7; *p* < 0.0001; *N* = 5). Tracking the number of eggs that eclosed to adults revealed that knocking down any of the three kinesin transcripts, khc, khc, or unc-104, significantly reduced adults (Fig. 9*G*, one-way ANOVA, *F* = 43.0; *p* < 0.0001; *N* = 5). To further assess the health and viability of the animals, the length, width, and area of third-instar larvae was examined; however, no significant differences we observed for kinesin 1 or 3 knockdown compared with OK6>dilp2-GFP controls (area: Fig. 9*H*, one-way ANOVA; length, *F* = 18.4; width, *F* = 2.9; area, *F* = 7.1; *N* = 10).

## Discussion

Here we conducted a targeted screen of six kinesin families, encompassing nine klps, to assess their role in the trafficking of NPs and the downstream impacts of kinesin disruption in motor axons. Upon screening six candidate kinesins involved in NP transport, we identified two kinesins, 1 and 3, that produced an aggregation phenotype. This finding aligns with prior studies demonstrating the essential role these kinesins serve in transport of other organelles and cargo including NPs, mitochondria, and SVs (Pilling et al., 2006; Kern et al., 2013; Lim et al., 2017; Liao et al., 2018; Gavrilova et al., 2024). Other kinesins, including Kif3C, KHC-73, KLP3A, CENP-meta (cmet), CENP-ana (cana), and Pavarotti, did not produce an obvious aggregation phenotype in the VNC or SNBs. This outcome was unsurprising, as Kif3C has been demonstrated as a microtubule-destabilizing factor required for axon regeneration rather than cargo transport (Gumy et al., 2013). Cana and cmet, *Drosophila* homolog of CENP-E, play key roles during stages of cell division, mediating processes such as chromosome alignment, spindle formation, and kinetochore assembly (Yen et al., 1992; Maia et al., 2007; Demirdizen et al., 2019). In mice, KLP3A mutations are understood to disrupt central spindle and cytokinesis in males, although somatic cells in *Drosophila* show insensitivity to loss of wild-type KLP3A (Williams et al., n.d.). On the other hand, KHC-73 has been shown to facilitate the routing of synaptic endosomes back to the soma (Liao et al., 2018). Finally, Pavarotti forms the centralspindlin complex, which plays a role during cytokinesis, but evidence for a role in neurons via interactions with kin-1 to regulate microtubule sliding and neurite outgrowth (Adams et al., 1998; Del Castillo et al., 2015). Both kinesins 1 and 3 exhibited a significant increase in the number of NP aggregates within SNBs, with kinesin 1 also showing a significant increase in the aggregate size. While there are at least 10 families of kinesins in *Drosophila*¸ to date our data support previous observations of kinesins 1 and 3 as pivotal motors of axonal NP trafficking (Mahajan-Miklos and Cooley, 1994; Miki et al., 2001; Hirokawa and Takemura, 2005; Hollenbeck and Saxton, 2005; Gavrilova et al., 2024). Our data also highlight numerous novel findings including kinesin disruptions led to significant changes in NP abundance at boutons, changes in synaptic morphology, synaptic capture, and release of NPs from synapses. Moreover, this study reveals a profound reduction in neuromuscular transduction, and excitation–contraction coupling (ECC) in kinesin 1 knockdowns, but not for kinesin 3.

Our investigation revealed that while both kinesins 1 and 3 are required for efficient anterograde transport, only kinesin 3, unc-104, plays a role in retrograde transport. Previously, retrograde transport of ANF filled DCVs was significantly disrupted in unc-104 mutants with no observable defects in retrograde transport of SVs and mitochondria (Barkus et al., 2008). One possibility for this kinesin-dependent role in retrograde transport lies in the complex of proteins associated with different organelles. Mitochondria have unique adapter proteins, e.g., milton, or from observations of Kif1C in mice, a kinesin 3 member, has shown its association with adaptor protein, HOOK3, whose binding enables dynein/dynactin recruitment, thereby facilitating retrograde transport (Abid Ali et al., 2025). Additionally, the phosphorylation of other adapter proteins huntingtin and JNK-interacting proteins are known to mediate the switch between retrograde and anterograde transport of cargo, which differentially interact with kinesin subtypes (Verhey and Rapoport, 2001; Colin et al., 2008). Recent reports are also generating data to support a more complex role for kinesins in both antero- and retrograde transport (Smith et al., 2023; Gavrilova et al., 2024). There is also evidence of functional interdependencies between kinesins and dynein/dynactin, with some evidence for a dual motor complex from the colocalization and direct physical interaction between kinesin light chains and the intermediate chain of dynein (Lee et al., 2018). Additional evidence suggests a regulatory feedback loops between kinesin and dynein following misexpression (Cianfrocco et al., 2015).

Intracellular organelle and protein aggregation in the nucleus and axons of neurons disrupt neuronal homeostasis, leading to cytotoxicity, neurodegeneration, and ultimately neuronal necrosis/apoptosis. Much of our current understanding of the downstream implications of protein aggregates is predicated based on neurodegenerative diseases like amyotrophic lateral sclerosis, Alzheimer's, Parkinson's, and Huntington's diseases (Weiss et al., 2012; Weiss and Littleton, 2016; Hana et al., 2024). Consequently, we explored the downstream ramifications of altered kinesin motor protein activity selectively in MNs. Initially, we investigated the effects of kinesin 1 (khc) and 3 (unc-104) knockdown on the growth and development of *Drosophila*. A morphological assessment of third-instar larvae did not reveal any significant changes in larval length, width, or area. This was surprising given previous reports from khc and klc mutant *Drosophila* larvae describe severe morphologically and physiologically impaired second and third instars (Saxton et al., 1991; Gho et al., 1992; Hurd et al., 1996; Kurd and Saxton, 1996). By tracking multiple developmental stages, from egg laying to adult eclosion, we aimed to determine whether impaired kinesin function leads to systemic physiological consequences of disrupted intracellular trafficking. Kinesin 1 knockdown significantly impaired larval development from first to third instar, showing >40% reduction in klc animals and 75+% reduction in khc. Previous reports from khc and klc mutant animals describe paralysis and stalled larval development during the second instar stage; however, larvae ultimately can progress to adulthood (Gho et al., 1992; Hurd et al., 1996; Kurd and Saxton, 1996). Collectively, kinesin 1 knockdown animals are severely impaired developmentally and functionally by the third-instar larvae stage, while minimal impacts were observed for kinesin 3 knockdown. To further dissect the molecular and cellular underpinnings of these effects, a thorough assessment of neuromuscular transduction was conducted.

Motor neurons (MNs) projecting from the VNC to body-wall muscles not only propagate electrical signals which dictate the patterned output regulating muscle contractile force/timing underlying larval movement but also transport cargo and numerous other cellular material necessary for neuronal growth, development, and homeostasis (Aponte-Santiago et al., 2020; Ormerod et al., 2022). The glutamatergic MNs-Ib (tonic) and MN-Is (phasic) subtypes within the VNC innervate body-wall muscles in highly stereotyped morphological varicosities with reproducible branching patterns and terminal-specific bouton numbers along the muscle surface (Peron et al., 2009). Knockdown of khc significantly reduced the extent of innervation along the muscle surface for MN-Ib and a concomitant reduction in the number of boutons. Interestingly, these effects were not observed for the MN-Is subtype. This difference could arise because of developmental effects, axonal–retraction/degeneration, or a failure of homeostatic plasticity to maintain synaptic structural integrity (Goel et al., 2019; Aponte-Santiago et al., 2020). Type Ib terminals have greater mitochondrial density and demand 2× the ATP per SV fusion event (Chouhan et al., 2010; Justs et al., 2022). Consequently, the metabolic demands of the Ib terminals may make them more susceptible to failure in transport of critical cargo like mitochondria (Lim et al., 2017). Noteworthy, our previous work demonstrated that MN-Is terminals precede MN-Ib terminals developmentally which could contribute to the morphological distinction observed herein (Aponte-Santiago et al., 2020). The definitive role of kinesins in axonal/growth cones pathfinding and proper development or maintenance of synaptic connections remains unclear (Pilling et al., 2006; Winding et al., 2016). A reduction in innervation length or bouton number for unc-104 was not observed; however, knockdowns of both kinesin 1 and 3 resulted in a reduction in BRP density at MN terminals (number of brp/bouton). A reduction in innervation length, bouton number, and BRP density correlated with a profound reduction in EJP amplitude, reported previously (Gho et al., 1992). The lack of effect of kinesin 3 knockdown was initially surprising given previous reports in the field where unc-104 bris, a hypomorphic allele, reduced EJP frequency (Zhang et al., 2017). However, the lack of observable morphological changes in synaptic structure does strongly correlate with a wild-type EJP amplitude. A reduction in BRP density in MN-Is terminals in unc-104 knockdown animals was observed, but previous reports suggest that this is insufficient to cause quantifiable changes in EJP amplitude as excess BRP exists at AZs (Akbergenova et al., 2018; Cunningham et al., 2022). These differences may arise from alterations in SV pools, which could be elucidated via increased stimulation frequency, to induce stress upon the system further, leading to enhanced synaptic rundown or depression.

Changes in EJP amplitude reflect alterations in synchronous NT release from SVs following depolarization (Katz and Miledi, 1967; Jan and Jan, 1976; Yoshihara and Littleton, 2002), which likely result from the structural changes at the NMJ. Fascinatingly, another striking observation from both kinesin 1 and 3 knockdown animals was the dramatic reduction in NP abundance at the synaptic varicosities along the surface of body-wall muscles. The cumulative impacts of axonal aggregates and trafficking deficits resulted in a 75% reduction in NP abundance and ∼90% reduction in NP moving through boutons. The effects were even more severe when examining synaptic capture and high-frequency–induced NP mobilization and release, where neither NP transiting from axons into bouton was observed nor was a significant drop in GFP fluorescence observed following 70 Hz stimulation (Rao et al., 2001; Levitan et al., 2007). Noteworthy, live imaging from boutons revealed no mobility in NP. Such a dramatic impact on NP release has been reported previously in the absence of calcium; however, to our knowledge, no other mechanism has been demonstrated to completely inhibit NP release from boutons (Shakiryanova et al., 2006). The lack of DCV mobility and release from boutons may be a consequence of inadequate ATP from insufficient mitochondrial trafficking, a completely incapacitated kinesin motor, or an NP that has uncoupled from the cytoskeleton.

Motivated linear crawling is the most highly stereotyped and orchestrated repetitive patterned behavior, produced from nearly identical contraction waves in body-wall muscle of *Drosophila* larvae (Hughes and Thomas, 2007; Heckscher et al., 2012). Muscles directly below the cuticle contract, then relax, to propagate peristaltic contraction waves from the posterior to the anterior of the larvae, predominately in abdominal segments (Ormerod et al., 2022). Our lab developed a robust force transducer assay to assess how changes in neuromuscular transduction manifest as alterations in ECC by examining changes in muscle force production with a 10 µN resolution (Ormerod et al., 2022). Knocking down khc severely impaired larval force production such that no observable force was generated until 20 Hz stimulation, and ∼80% reduction in muscle force production was observed. The reduction in muscle force production is likely a product of the impaired neuromuscular transduction we observed in our EJP recordings, as we did not observe any dramatic changes in muscle ultrastructure. This correlates with our previous work that ECC is most strongly coupled to neuromuscular transduction (Ormerod et al., 2022). Altered synapse morphology, neuromuscular transduction, and ECC in khc knockdowns all correlated with the deficits in larval crawling observed. The reduction in larval crawling velocity, linearity, and linear displacement observed in our locomotory is consistent with previous reports or larval paralysis during the second- and third-instar stages, as well as *C. elegans* where knock-out of Unc-116 (KHC) caused a suppression of overall crawling movement (Gindhart et al., 1998; Gavrilova et al., 2024). Knock-out mice of KIF5b (Kinesin 1) also show clear impairments in rhythmic locomotory behavior (Cromberg et al., 2019).

In summary, our data demonstrate which of the kinesin proteins are critical regulators of intracellular trafficking and reveal that disruption in the expression of kinesins 1 and 3 led to severe downstream implications, even when altered solely in MNs. Downstream impacts of kinesin disruption caused significant changes in NP abundance at boutons and changes in synaptic morphology, including innervation length, bouton number, and AZ composition. Spinning-disc confocal microscopy enabled subquantal level resolution of NP at boutons, revealing a profound impact on NP mobilization and release from boutons. Collectively, these disruptions manifest as significant reductions in neuromuscular transduction, ECC, and larval crawling in kinesin 1 knockdowns, but not for kinesin 3. Additionally, the unique role of kinesin 3 in retrograde signaling may provide evidence for cargo-specific motors to facilitate unique transport requirements. Taken together we have not only identified which kinesins are critically involved in organelle trafficking but also revealed critical disruptions in cellular morphology, function, physiology, and behavior in genetically disrupted animals.

## Synthesis

Reviewing Editor: Deanna Smith, University of South Carolina Decisions are customarily a result of the Reviewing Editor and the peer reviewers coming together and discussing their recommendations until a consensus is reached. When revisions are invited, a fact-based synthesis statement explaining their decision and outlining what is needed to prepare a revision will be listed below. The following reviewer(s) agreed to reveal their identity: Atulya Iyengar. Note: If this manuscript was transferred from JNeurosci and a decision was made to accept the manuscript without peer review, a synthesis may not be available.

The reviewers and I find that there are some useful new findings in this study. However, there are issues that need to be addressed in the resubmission. Please make it clear which findings are replicative of other work, and which are new experimental results, as many of the basic points are already established in the field, such as findings that Kinesin 1 and Kinesin 3 are critical for vesicle trafficking (Kinesin 1 - Pilling et al., 2006 MBoC; Kinesin 3 - Kern et al., 2013 Genetics, Liao et al., 2018 PloS Genetics). In light of this, please emphasize new findings reported in your manuscript and provide rationale for the choice of kinesins used in this study. In the introduction please be clear about the aims of your study. Do not highlight new text in red, as this is very difficult to read.

## Author Response

Synthesis Statement for Author (Required):

Author responses to review comments are found in red.

Please note: the reviewers requested for our changes: "Do not highlight new text, as this is very difficult to read, but use italics instead". However, the email we received from eNeuro indicated "Your revision must include the manuscript with new text indicated in a bold or highlighted font to aid the re-review of the manuscript" Our previous track-changes format aligned with the requests from J. Neurosci. ⇒ The changes in the word document here were italicized and bolded, we hope that is ok.

The reviewers and I find that there are some useful new findings in this study. However, there are some issues that need to be addressed in the resubmission.

1. Please make it clear which findings are replicative of other work and which are new experimental results, as many of the basic points are already established in the field, such as findings that Kinesin 1 and Kinesin 3 are critical for vesicle trafficking (Kinesin 1 - Pilling et al., 2006 MBoC; Kinesin 3 - Kern et al., 2013 Genetics, Liao et al., 2018 PloS Genetics). In light of this, please emphasize new findings reported in your manuscript and provide rationale for the choice of kinesins used in this study.

To address our choice of kinesins we have added the following lines to the results section:

Lines 221-225: We conducted a targeted genetic screen using all available RNAi lines against kinesins from the Blooming Drosophila Stock Center. We were able to acquire RNAi lines to knock-down 9 different kinesin proteins including 6 different families of kinesins: kinesin 1 (kinesin heavy chain, kinesin light chain), kinesin 2 (Kif3C), kinesin 3 (unc-104, khc-73), kinesin 4 (Klp3A), kinesin 6 (pav), and kinesin 7 (cmet and cana).

We have added references to all three papers (Pilling et al, 2006; Kern et al, 2013; and Lioa et al 2018) in both the introduction, results and discussion sections:

Lines 77-79, lines 218-220, lines 225-227, and lines 418-420.

There are several instances throughout the paper where we highlight previous works and the importance of kinesin 1 and 3 in organelle trafficking:

Introduction: Lines 74-88.

Results: lines 218-220: "Two families of kinesins, 1 and 3, have been identified and shown to be critical in regulating the trafficking of organelles like SVs, NPs, and mitochondria, and some evidence exists for kinesins 2, 4 in neuronal trafficking" Dicussion: lines 418-420: This finding aligns with prior studies demonstrating the essential role these kinesins (kinesins 1 and 3) serve in transport of other organelles and cargo including: NPs, mitochondria, and SVs 25-27,47,48.

Lines 433-435: While there are at least 10 families of kinesins in Drosophila¸ to date our data supports previous observations of kinesin 1 and 3 as pivotal motors of axonal NP trafficking 11,12,62-64.

To specifically highlight which findings are novel, we have added the following to the discussion: lines 435-440:

Our data also highlight numerous novel findings including: kinesin disruptions led to significant changes in neuropeptide abundance at boutons, changes in synaptic morphology, synaptic capture and release of NPs from synapses. Moreover, this study reveals a profound reduction in neuromuscular transduction, and excitation-contraction coupling in kinesin 1 knockdowns, but not for kinesin 3.

2. In the introduction please be clear about the aims of your study. Do not highlight new text, as this is very difficult to read, but use italics instead We thank the reviewers/editor for this suggestion. We have made the following changes to the introduction to more clearly state our research aims:

Lines 89-92: Here we conducted a targeted genetic screen using RNAi to knockdown 6 families of kinesin proteins in Drosophila which aims to identify those involved in neuropeptide (NP) trafficking in motor axons. In this study we also aim to examine the downstream molecular, cellular, and physiological impacts of disrupted motor protein trafficking.
